# Tongue Sole CD209: A Pattern-Recognition Receptor that Binds a Broad Range of Microbes and Promotes Phagocytosis

**DOI:** 10.3390/ijms18091848

**Published:** 2017-09-04

**Authors:** Shuai Jiang, Li Sun

**Affiliations:** 1 Key Laboratory of Experimental Marine Biology, Institute of Oceanology, Chinese Academy of Sciences, Qingdao 266071, China; sjiang@qdio.ac.cn; 2 Laboratory for Marine Biology and Biotechnology, Qingdao National Laboratory for Marine Science and Technology, Qingdao 266071, China

**Keywords:** CD209, lectin, *Cynoglossus semilaevis*, microbial recognition, phagocytosis

## Abstract

CD209 is an immune receptor that plays an important role in the initiation of innate immunity and activation of adaptive immunity in mammals. However, much less is known about the immunological function of CD209 in lower vertebrates. In the present study, we examined the immune effect of a CD209 homologue (CsCD209) from the teleost fish tongue sole *Cynoglossus semilaevis*. CsCD209 possesses a lectin domain that shares high levels of similarity with the lectin domains of human and mouse CD209. CsCD209 expression was most abundant in kidney and blood and was significantly upregulated during bacterial infection. CsCD209 exhibited a subcellular localization mainly on the cell surface of myelomonocytes. Recombinant CsCD209 displayed apparent binding capacities to a broad range of bacteria and fungi, and significantly promoted the phagocytosis of the bound bacteria by *C. semilaevis* leukocytes. Collectively, the results indicate that teleost CD209 serves as a pattern recognition receptor that exerts an influence on the phagocytosis process during pathogen infections.

## 1. Introduction

Innate immune sensing plays an essential role in the initiation of innate immunity and activation of adaptive immunity in vertebrates [1,2]. The innate immune system utilizes a limited number of pattern recognition receptors (PRRs) to sense pathogen-associated molecular patterns (PAMPs) present exclusively in bacteria, fungi, and viruses [3,4]. Several types of PRRs, such as Toll-like receptors (TLRs) and Nod-like receptors (NLRs), have been reported, which show different microbial recognition activities during the invasion of pathogens [5].

CD209, also known as dendritic cell-specific intercellular adhesion molecule-3-grabbing non-integrin (DC-SIGN), is a member of the C-type lectin family. It functions as an important PRR in immune defense and microbial pathogenesis in mammals [6,7]. In humans, CD209 is mainly present on the surface of macrophages and certain types of dendritic cells (DCs) [8]. In macrophages, CD209 can bind to mannosyl glycans on viruses, bacteria, and fungi, which in turn promote macrophage phagocytosis [9,10]; CD209 expressed in DCs was mainly involved in the modulation of cellular interactions as well as pattern recognition [11,12]. CD209 has been proven to play important roles in the immune modulation during pathogen infection [13]. It was reported to bind *Candida albicans* and serve as a phagocytic receptor in monocyte-derived DCs [11]. CD209 also exhibited high binding capacities towards human immunodeficiency virus (HIV) and the hepatitis C virus [14,15]. Besides functioning as a PRR, CD209 is able to initiate innate immune response by cross talking with TLR-mediated signaling pathway [16].

In fish, studies on CD209 and DCs are far from clear. A type of DC-like leukocytes with antigen-presenting functions was reportedly identified in zebrafish *Danio rerio*, which expressed DC function-associated genes [17], and a zebrafish CD209 homologue was found to be involve in the modulation of adaptive immunity [18]. In rainbow trout *Oncorhynchus mykiss*, CD208/lysosomal associated membrane protein 3 and CD209 were studied for the candidates of the biomarkers in DC characterization [19,20]. In the present study, with an aim to elucidate the immune function of fish CD209, we examined the expression and distribution of tongue sole (*Cynoglossus semilaevis*) CD209 (CsCD209) in tissues and cells, and investigated the immunological property of CsCD209 as a PRR.

## 2. Results

### 2.1. Sequence Analysis of CsCD209

**Figure 1:**
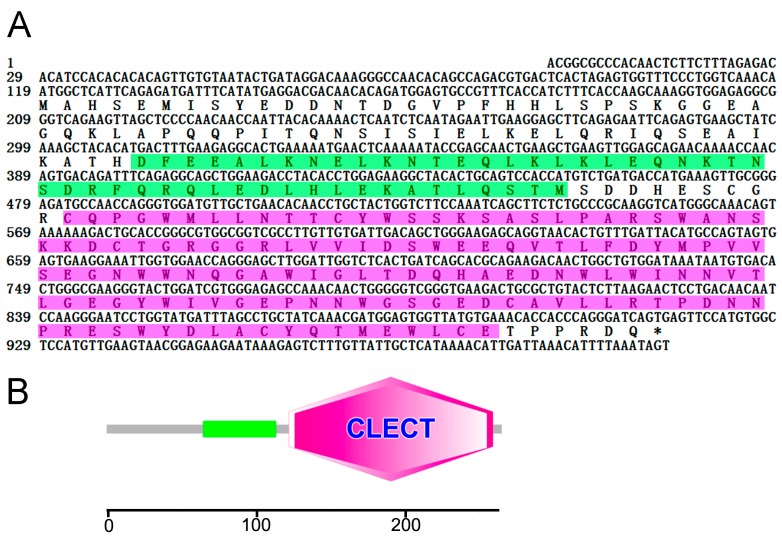
Sequence analysis of CsCD209. (**A**) The nucleotides and amino acids are numbered along the left margin. The translation stop codon is labeled with asterisk. The amino acid sequence of coiled-coil region and the lectin domain analyzed by SMART (http://smart.embl.de/) was boxed with green and pink respectively; (**B**) The schematic of protein motifs of CsCD209. The coiled-coil region (65–122) is indicated with green rectangle, and the lectin domain (122–259) is indicated with pink hexagon.

**Figure 2:**
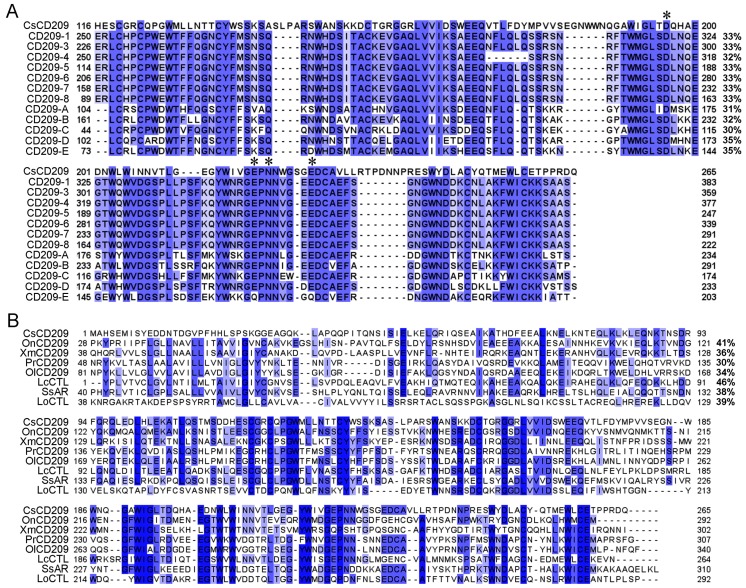
Multiple alignment of CsCD209 with other CD209/lectin proteins. (**A**) Multiple alignment of the lectin domains of CsCD209 and CD209 from *Homo sapiens* (CD209-1, -3, -4, -5, -6, -7 and -8) and *Mus musculus* (CD209-A, -B, -C, -D and -E) that share 30–35% similarities with CsCD209 (right side of the alignment). The conserved amino acid residues involved in mannosylated glycan interaction are indicated with asterisks; (**B**) Multiple alignment of full-length CsCD209 homologues from teleost fishes. OnCD209: *Oreochromis niloticus* CD209, XmCD209: *Xiphophorus maculates* CD209, PrCD209: *Poecilia reticulate* CD209, OlCD209: *Oryzias latipes* CD209, LcCTL: *Larimichthys crocea* C-type lectin, SsAR: *Salmo salar* asialoglycoprotein receptor, LoCTL: *Lepisosteus oculatus* C-type lectin. In both (**A**) and (**B**), dots denote gaps introduced for maximum matching. The blue shadow color indicates a similarity between sequences, with darker shades meaning greater similarities.

**Figure 3:**
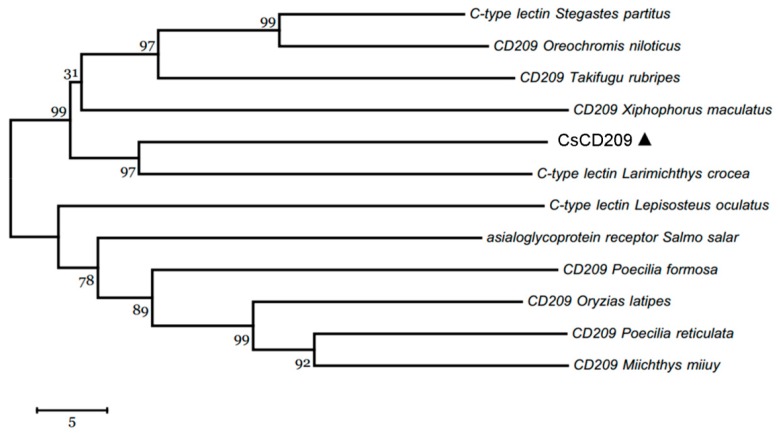
Phylogenetic analysis of CsCD209 and other CD209 proteins. The phylogenetic tree was constructed with MEGA 6.0 software (http://www.megasoftware.net/) using the neighbor-joining method. CsCD209 was marked by triangle. Numbers beside the internal branches indicate bootstrap values based on 1000 replications. The 5 scale indicates the genetic distance.

CsCD209 is composed of 265 amino acids and has a theoretical molecular weight of 30306.55 Da and a predicted isoelectric point of 4.72 (Figure 1A). CsCD209 contains a coiled-coil region (65–122 aa) and a carbohydrate recognition domain (CRD, 122–259 aa) (Figure 1B). CsCD209 shares high levels of similarity with human and mouse CD209 in the lectin domain, especially for the conserved residues, such as Asp196, Glu220, Asn222, and Glu228, known to be involved in mannosylated glycan binding (Figure 2A). CsCD209 also exhibited significant amino acid sequence similarity with CD209 homologues from other teleost fishes (Figure 2B). Phylogenetic analysis showed that CsCD209 and a C-type lectin of *Larimichthys crocea* formed a cluster, which was branched off from the group formed by CD209/C-type lectins from *Xiphophorus maculates*, *Takifugu rubripes*, *Oreochromis niloticus* and *Stegastes partitus* (Figure 3).

### 2.2. Three-Dimensional Structure Characteristics of CsCD209

**Figure 4:**
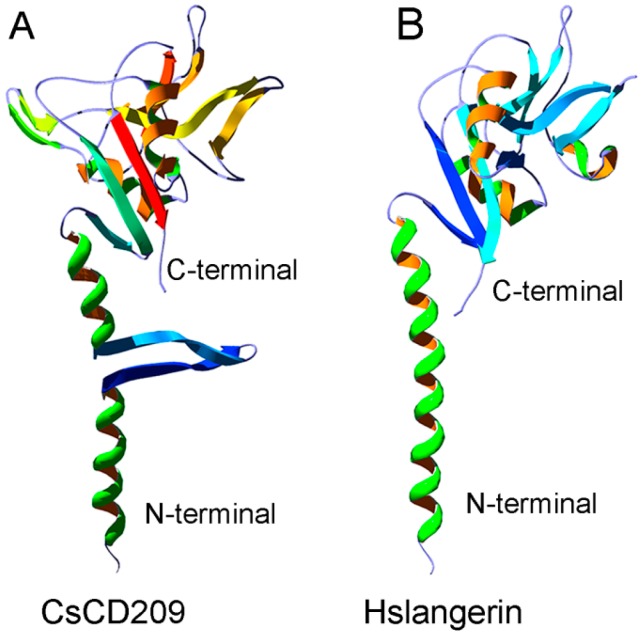
Comparison of the three-dimensional structures between CsCD209 and human langerin. (**A**) The three-dimensional model of CsCD209 was produced by homologous modeling from the SWISS-MODEL program (http://www.expasy.org/swissmod/SWISS- MODEL.html); (**B**) The crystal structure of human langerin (PDB: 3KQG). Hs: *Homo sapiens*. The protein structure was indicated by rainbow-spectrum coloring by PyMOL (http://www.pymol.org/).

The potential three-dimensional structure of CsCD209 was predicted by the automated SWISS-MODEL homology modeling pipeline based on the template protein langerin (PDB: 3KQG), which is a human C-type lectin (Figure 4A). CsCD209 contained a *N*-terminal coiled-coil region and a C-terminal CRD region. The coiled-coil region of CsCD209 was predicted to adopt α-helices and two β-sheets, while the corresponding region of langerin formed only α-helices without β-sheet. In the lectin domain, CsCD209, like langerin, exhibited two α-helices and six β-sheets, which formed similar three-dimensional structures (Figure 4B).

### 2.3. Expression of CsCD209 in Fish Tissues

**Figure 5:**
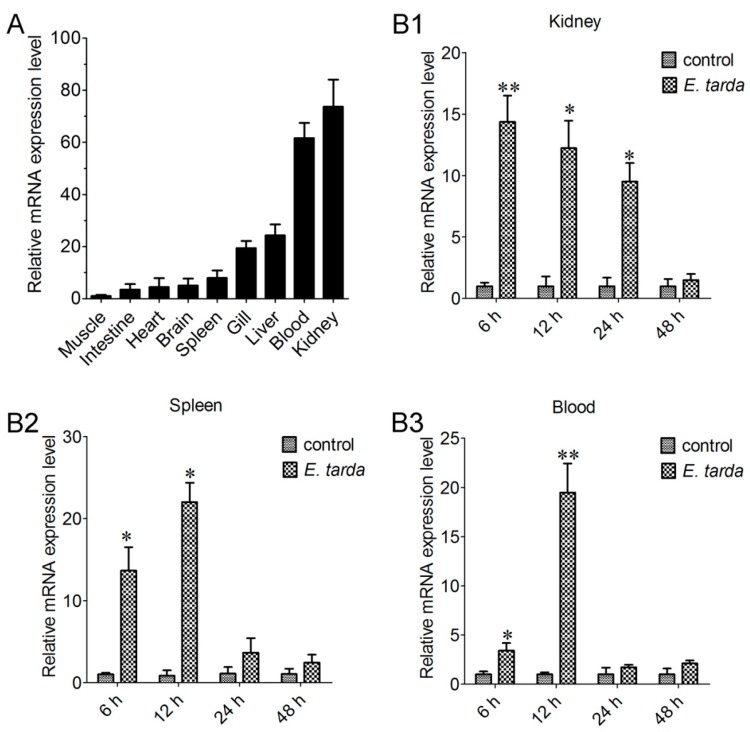
CsCD209 expression in fish tissues under physiological and pathological conditions. (**A**) CsCD209 expression in the muscle, intestine, heart, brain, spleen, gill, liver, blood and kidney of tongue sole was determined by qRT-PCR. For comparison, the expression level of CsCD209 in muscle (the lowest expression level) was normalized as 1; The expression of CsCD209 in kidney (**B1**); spleen (**B2**) and blood (**B3**) during *Edwardsiella tarda* infection was determined by qRT-PCR at various time points. In each case, the expression level of the control fish was normalized as 1. Data are the means of three independent experiments and shown as means ± standard error of the mean (SEM). * *p* < 0.05, ** *p* < 0.01.

qRT-PCR analysis showed that CsCD209 expression was most abundant in kidney and blood and least abundant in muscle under normal physiological conditions. Compared with the CsCD209 expression level in muscle, the CsCD209 expression levels in the intestine, heart, brain, spleen, gill, liver, blood and kidney were 3.7-, 6.1-, 6.9-, 8.3-, 19.6-, 24.1-, 63.3- and 72.5-fold higher, respectively (Figure 5A). When tongue sole fish were infected with the bacterial pathogen *Edwardsiella tarda*, the expression levels in kidney were significantly up-regulated, with the highest level occurring at 6 hour post infection (hpi), and gradually decreased at 12, 24 and 48 hpi (Figure 5B1). CsCD209 expression in spleen induced by *E. tarda* infection significantly increased at 6 hpi and peaked at 12 hpi, and fell back to the normal level at 24 and 48 hpi (Figure 5B2). The expression level in blood was significantly up-regulated at 6 hpi, with the highest expression level occurring at 12 hpi, and was decreased at 24 and 48 hpi (Figure 5B3).

### 2.4. Expression of CsCD209 in Head Kidney Leukocytes and Blood

**Figure 6:**
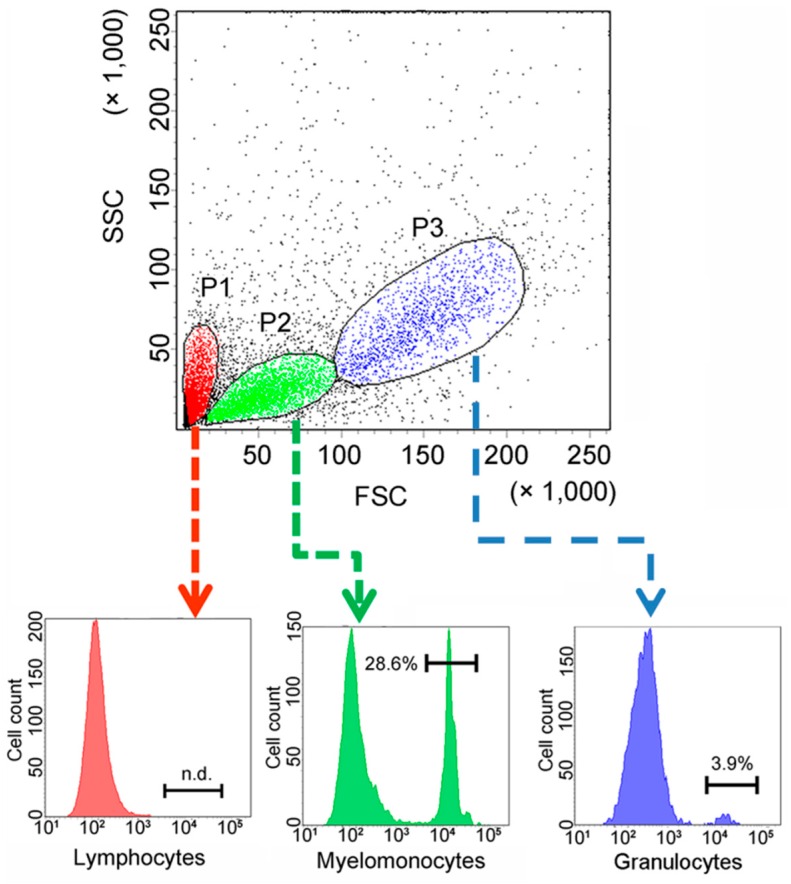
Cell-specific expression of CsCD209 determined by flow cytometry. Single cell suspension was prepared from head kidney and labeled with antibody against recombinant CsCD209 (rCsCD209) and Alexa Fluor 488-labeled Goat anti-mouse IgG. Dot plot (upper panel) demonstrates the distribution of cells in head kidney by light scatter: lymphocytes (red), myelomonocytes (green), and granulocytes (blue). Histogram shows the expression of CsCD209 in lymphocytes (lower left), myelomonocytes (lower middle), and granulocytes (lower right).

**Figure 7:**
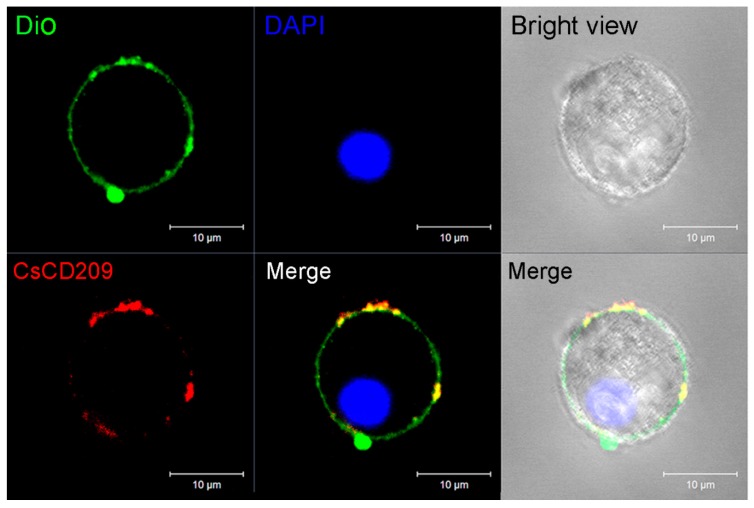
Confocal microscopic analysis of CsCD209 distribution in leukocytes from head kidney. The distribution of CsCD209 was indicated by Alexa Fluor 594 (red color)-labeled goat anti-mouse IgG, the cell membrane was stained by DiO (green color), and the nucleus was stained with 4′-6-diamidino-2-phenylindole (DAPI, blue color). Bar: 5 μm.

To facilitate functional study, recombinant CsCD209 (rCsCD209) and polyclonal antibody against rCsCD209 were prepared (Figure S1). With the antibody, CsCD209 expression and distribution in head kidney leukocytes were determined by flow cytometry as well as confocal microscopy. The flow cytometry based on the forward scatter (FSC) and side scatter (SSC) revealed that there were three cell populations, designated P1, P2 and P3, in head kidney leukocytes. According to the flow cytometric characteristics, cells in P1, P2 and P3 populations, which were similar to those observed in zebrafish head kidney leukocytes [21], were categorized as lymphocytes, myelomonocytes and granulocytes, respectively. Flow cytometry showed that CsCD209 expression was detected mainly on myelomonocytes (28.6%), much less on granulocytes (3.9%), and barely on lymphocytes (Figure 6). For microscopy, anti-CsCD209 antibody and Alexa Fluor 594-labeled secondary antibody were applied to reveal the localization of CsCD209. It was found that CsCD209 (red) was co-localized with DiO-stained membrane (green), suggesting distribution of CsCD209 in the cell membrane (Figure 7). The CsCD209 signal was barely observed in the cytoplasm. Western blot showed that CsCD209 was detected in serum (Figure S2), suggesting the existence of soluble form of CsCD209.

### 2.5. Microbial Binding Activity of rCsCD209

**Figure 8:**
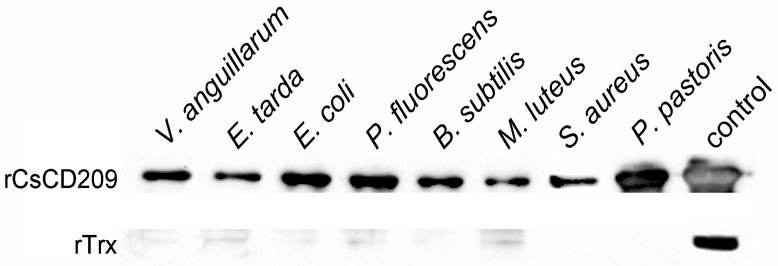
Western blot analysis of the microbial binding activity of rCsCD209. *V. anguillarum*, *E. tarda*, *E. coli*, *P. fluorescens*, *B. subtilis*, *M. luteus*, *S. aureus* and *P. pastoris* were incubated with rCsCD209 or rTrx. The bound proteins were separated by SDS-PAGE and immunoblotted with anti-His monoclonal antibody. The purified rCsCD209 and rTrx were loaded as the control.

To examine its potential to interact with microorganisms, rCsCD209 or the control protein recombinant thioredoxin (rTrx), was incubated with the Gram-negative bacteria *Vibrio anguillarum*, *E. tarda*, *Escherichia coli*, and *Pseudomonas fluorescens*, the Gram-positive bacteria *Bacillus subtilis*, *Micrococcus luteus* and *Staphylococcus aureus*, and yeast *Pichia pastoris*. Binding of the protein to the microbes was subsequently determined by Western blot. The results showed that compared with rTrx, rCsCD209 exhibited apparent binding activities to all examined species of bacteria and yeast (Figure 8). Furthermore, flow cytometry analysis showed that when rCsCD209 was pre-incubated with mannose or mannan, the binding activity of rCsCD209 to *E. tarda was* significantly reduced (Figure S3A,B).

### 2.6. Effect of rCsCD209 on Phagocytosis Towards Bacteria

**Figure 9:**
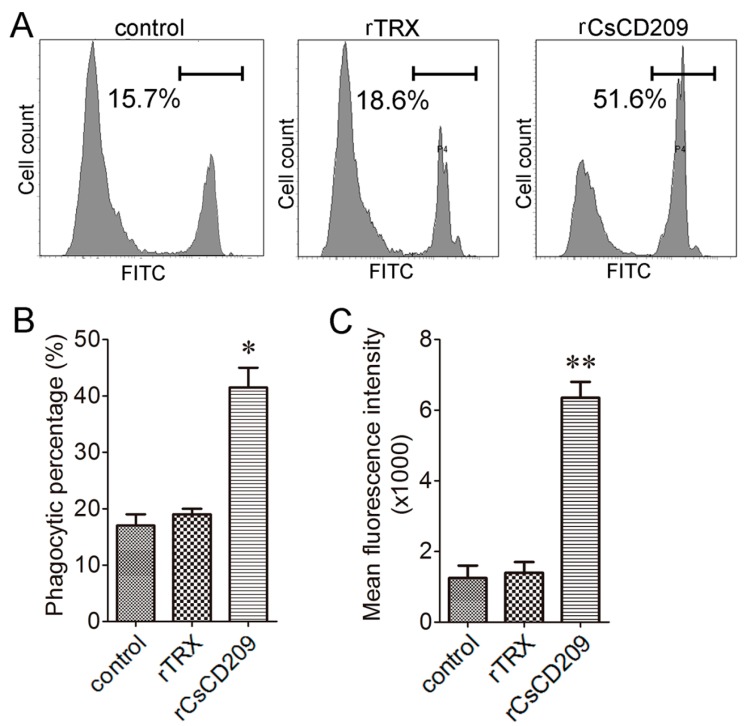
rCsCD209 enhanced phagocytosis of bacteria. Head kidney leukocytes were incubated with fluorescein isothiocyanate (FITC)-labeled *Edwardsiella tarda* that had been pre-incubated with rCsCD209 or rTrx. Phagocytosis activity was measured by flow cytometry (**A**); Phagocytic percentage (**B**) and phagocytic index (**C**) were statistically calculated. Results are means ± SEM (*n* = 5), *p < 0.05, ** *p* < 0.01.

To examine whether CsCD209-bacteria interaction affected the phagocytosis of the bacteria by *C. semilaevis* immune cells, *E. tarda* was incubated with or without rCsCD209 before encountering head kidney leukocytes, and phagocytosis was subsequently determined by flow cytometry (Figure 9A). The results showed that the phagocytic percentage of leukocytes towards *E. tarda* was significantly increased after rCsCD209 preincubation (*p* < 0.05), whereas there was no significant change after rTrx treatment (Figure 9B). Moreover, the phagocytic index of the leukocytes preincubated with rCsCD209 was significantly higher than that of the control and the leukocytes preincubated with rTrx (*p* < 0.01) (Figure 9C).

## 3. Discussion

In mammals, CD209 is a type II membrane protein that contains a C-terminal carbohydrate recognition domain (CRD) and a coiled-coil neck region in the extracellular domain [22]. CRD is responsible for the pattern recognition towards different glycans, while the neck region is necessary for oligomerization [23]. The formation of multimeric complexes and the conformational change of CD209 increase the binding avidity to glycan ligands [24]. The amino acid sequence of CsCD209 shares moderate identities with the CD209 isoforms of human and mice, especially in the CRD region, suggesting a conserved function of CD209 between higher and lower vertebrates. The three-dimensional structure model of CsCD209 exhibited a strong similarity to langerin, a lectin-type immune receptor with structure characteristics, including the neck region and C-terminal CRD, similar to that of CD209 [25]. However, it was noteworthy that, unlike langerin, CsCD209 exhibited two β-sheets in the neck region, suggesting a conformational variation in the three-dimensional structure of fish CD209.

In humans, CD209 is expressed on several types of macrophage populations that produce pro-inflammatory cytokines and thus play an essential role in the activation of inflammatory immune response [26,27]. More pronouncedly, CD209 is expressed on the surface of immature DCs and mature DCs under the modulation of interleukin-4 [28]. DCs have been proved to be of fundamental importance in orchestrating mammalian immune response. However, much less is known about the DCs in lower vertebrates. In zebrafish, a type of DC-like leukocytes with antigen-presenting functions was identified, which expressed DC function-associated genes [17]. In addition, a CD209 homologue was also identified in zebrafish, which was involved in adaptive immunity activation, including T cell activation, IgM production, and bacterial vaccination-elicited immune protection [18]. In rainbow trout, the homologue of the human dendritic cell marker CD208/lysosomal associated membrane protein 3 was found to be constitutively expressed in head kidney macrophages, and up-regulated after infection with viral and bacterial pathogens, which is important to the antigen presentation investigation in teleost fish [20]. In addition, CD209 combined with CD83, CXC chemokine receptor-4 (CXCR-4) and CC chemokine receptor-7 (CCR-7) were used as biomarker candidates of DCs [19]. Recently, a CD8α^+^ MHC II^+^ DC-like subpopulation was identified in the rainbow trout skin, showing phenotypical and functional similarities of semimature DCs, thus supported the hypothesis of a common origin for all mammalian cross-presenting DCs [29]. In our study, we found that CsCD209 mRNA was most abundant in kidney and blood and significantly augmented by bacterial infection, and that CsCD209 protein occurred on the surface of a certain subpopulation of myelomonocytes in head kidney leukocytes and on a very small population of granulocytes. In addition, the CsCD209 protein was also detected in blood, suggesting that CsCD209 may, like human CD209 which is known to exist as both membrane and soluble proteins [30], be generated in both membrane and soluble forms. These results indicate that CsCD209 may play a role in pathogen-induced immunity.

CD209 is a C-type lectin belonging to a large and diverse lectin family found in a wide range of vertebrates [31,32]. It mainly functions as a PRR through its CRD, which exhibits an extensive microbial binding activity to mannosylated glycans on pathogens [33]. For example, CD209 was reported to bind directly to mycobacteria-specific lipoglycan lipoarabinomannan presented on the surface of *Mycobacterium tuberculosis*, whereby mediating bacterial entry into DCs [34]; CD209 recognized and bound highly mannosylated envelope glycoprotein gp120 from HIV and promoted viral infection of CD4^+^ T lymphocytes [35]. In our study, we found that rCsCD209 exhibited an extensive binding activity to various microbes including Gram-negative and Gram-positive bacteria as well as fungi, indicating that CsCD209 can serve as a PRR with a broad microbial recognition range, which may facilitate the initiation of immune response during pathogen invasion.

Phagocytosis plays a vital role in innate immune defense and activation of adaptive immunity [36,37]. In mammals, phagocytes have evolved a number of receptors to facilitate the phagocytic process [38], for example, human CD209 functioned as an immune receptor for *Yersinia pestis* and promoted phagocytosis by DCs [39]. It could also bind specifically to the core LPS of *E. coli* K12, promoting bacterial adherence and phagocytosis [40]. DCs expressing CD209 were reported to capture the hepatitis C virus by specific binding to the envelope glycoprotein E2, and efficiently transinfected adjacent human liver cells [41]. In the teleost fish sea bass, macrophages and neutrophils possess potent phagocytic capacities against *Photobacterium damselae* infection [42]. In the case of tongue sole, we found that preincubation of *E. tarda* with rCsCD209 significantly enhanced phagocytosis of the bacteria by tongue sole head kidney leukocyte, suggesting that rCsCD209 promoted the intracellular uptake of the bound bacteria. This result, together with the observation that *E. tarda* upregulated the expression of CsCD209, indicates that CsCD209 probably acts as an innate immune factor that contributes to the clearance of invading pathogens.

In conclusion, the present study reveals that CsCD209 serves as a PRR with microbial recognition activity against a wide range of bacteria and fungi, and that CsCD209 modulates the phagocytosis of pathogens by host immune cells. These results suggest an important role of fish CD209 in antimicrobial immune defense.

## 4. Materials and Methods

### 4.1. Fish

Clinically healthy tongue sole were purchased from a commercial fish farm in Shandong Province, China, and maintained at 20 °C in aerated seawater. The fish were acclimatized in the laboratory for 2 weeks and verified to be clinically healthy. All animal-involving experiments of this study were approved by the Ethics Committee of Institute of Oceanology, Chinese Academy of Sciences as reported previously [43].

### 4.2. Bacterial Strains and Culture Conditions

Vibrio anguillarum, Edwardsiella tarda and Pseudomonas fluorescens were grown in Luria–Bertani (LB) broth at 28 °C for 12 h with shaking at 180 rpm. Escherichia coli, Staphylococcus aureus, Micrococcus luteus and Bacillus subtilis were grown in LB broth at 37 °C for 8 h with shaking at 220 rpm. Pichia pastoris was grown in yeast extract peptone dextrose (YPD) medium at 30 °C for 24 h with shaking at 220 rpm. All microbes were grown to mid-log phase and harvested by centrifugation at 6000× *g* for 15 min, followed by washing three times with PBS (pH 7.2).

### 4.3. Sequence Analysis

The BLAST algorithm (http://www.ncbi.nlm.nih.gov/blast) and the Expert Protein Analysis System (http://www.expasy.org) were performed to analyze the amino acid sequence. Domain search was performed with the simple modular architecture research tool (SMART) version 4.0 (http://www.smart.emblheidelberg.de/). The potential three-dimensional structure was established using the SWISS-MODEL prediction algorithm (http://swissmodel.expasy.org/) and displayed by Deepview/Swiss-Pdb Viewer version 4.0. The ClustalW multiple alignment program (http://www.ebi.ac.uk/clustalw/) was performed to create the multiple sequence alignment. Phylogenetic analysis was performed with the neighbor-joining algorithm of MEGA 5.0.

### 4.4. Quantitative Real Time Reverse Transcription-PCR (qRT-PCR)

#### 4.4.1. qRT-PCR Analysis of CsCD209 Expression in Different Fish Tissues under Normal Physiological Conditions

Kidney, blood, intestine, gill, brain, muscle, heart, spleen, and liver were taken aseptically from five tongue soles (average 13.6 g). Total RNA was extracted and incubated with DNase I to digest genomic DNA using the EZNA Total RNA Kit (Omega, Norcross, GA, USA) according to the manufacturer‘s instructions. Total RNA (1 μg) was used for cDNA synthesis with Superscript II reverse transcriptase (Invitrogen, Carlsbad, CA, USA). The primers F1 (5′-GATTACATGCCAGTAGTGAGTGAAGG-3′) and R1 (5′-CCTTGGATTGTTGTCAGGAGTTC-3′) were used for amplification. The qRT-PCR program was performed as follows: 94 °C for 2 min, followed by 40 cycles at 94 °C for 15 s, 55 °C for 15 s, and 57 °C for 30 s. qRT-PCR was carried out in an Eppendorf Mastercycler (Eppendorf, Hamburg, Germany) using the SYBR ExScript qRT-PCR Kit (Takara, Dalian, China). The expression levels of CsCD209 mRNA in different fish tissues were analyzed using comparative threshold cycle method (2^−^^ΔΔ*C*t^) with β-actin as an internal reference [44]. The primers designed for β-actin were as follows: forward primer: 5′-GCACGGTATTGTGACCAACTGG-3′, reverse primer: 5′-CAGGGGAGCCTCTGTGAGC-3′. The experiment was performed in triplicate, each time with five fish.

#### 4.4.2. qRT-PCR Analysis of CsCD209 Expression in Different Fish Tissues during Pathogen Infection

*E. tarda* was cultured in LB broth at 28 °C to OD_600_ 0.6. The cells were washed with PBS (pH 7.2) and resuspended in PBS (pH 7.2) to 1 × 10^6^ colony-forming units (CFU)/mL. Tongue sole were randomly divided into three groups, and intraperitoneally injected with 50 μL *E. tarda*. The control group were injected with PBS. Kidney, spleen and blood were aseptically taken from the fish at 6, 12, 24, and 48 h post infection. qRT-PCR analysis of CsCD209 expression in different fish tissues was performed as described above. β-actin was used as the internal reference for kidney and blood, and ribosomalprotein L18 (RPL18) was selected as the internal reference for spleen [44]. The primers designed for RPL18 were as follows: forward primer: 5′-GAACCCTACCCCTCCTCTGT-3′, reverse primer: 5′-TACGAGAGTCGTAACGCAGC-3′. All experiments were performed in triplicate, each with five fish.

### 4.5. Prokaryotic Expression and Purification of Recombinant Proteins

The coding sequence of CsCD209 was amplified by PCR with gene-specific primer F2 (5′-ATGGCTCATTCAGAGATGATTTCATATGAGG-3′) and R2 (5′-TCACTGATCCCTGGGTGGTG-3′). The PCR amplification was performed as follows: 5 min at 95 °C, 30 s at 94 °C and 1 min at 72 °C, for 28 cycles. The PCR product was separated by agarose gel electrophoresis and was cloned into PMD19-T simple vector (Takara, Dalian, China) followed by sequencing, and was verified with target CsCD209 coding sequence. The coding sequence was inserted into the Nde I and Xho I sites of the pET-30a (+) (Novagen, Darmstadt, Germany) expression vector. The recombinant plasmid was isolated using MiniBEST plasmid purification kit (Takara, Dalian, China), and was transformed into *Escherichia coli* Transetta (DE3) (Transgen, Beijing, China) competent cells. The transformant was cultured in liquid LB broth containing 100 mg·L^−1^ kanamycin to an OD_600_ of 0.4–0.6, and isopropyl β-d-1-thiogalactopyranoside (AiKB, Qingdao, China) was added to the culture at the final concentration of 0.1 mM. The culture was continued at 16 °C for 12 additional hours, and rCsCD209 was purified with a Ni^2+^ chelating Sepharose column (Roche, Mannheim, Germany). The protein was desalted by extensive dialysis against PBS (pH 7.2), and Triton X-114 was used to remove endotoxin as previously reported [45]. rCsCD209 was subjected to 15% sodium dodecyl sulfate (SDS)-polyacrylamide gel electrophoresis (PAGE) and visualized by Coomassie brilliant blue R-250 staining. The protein concentration was quantified with an Enhanced BCA protein assay kit (Beyotime, Shanghai, China). Recombinant thioredoxin (rTrx) protein was prepared as described previously [44]. Briefly, *E. coli* BL21 (DE3) was transformed with pET32a (Novagen, Darmstadt, Germany), and the transformant was cultured in LB medium at 37 °C to an OD_600_ of 0.4–0.6. Isopropyl-β-d-thiogalactopyranoside was added to the culture at the final concentration of 0.4 mM. The culture was continued at 30 °C for 4 additional hours, and rTrx was purified with a Ni^2+^ chelation Sepharose column (Roche, Mannheim, Germany). The protein was desalted by extensive dialysis against PBS (pH 7.2). rTrx was subjected to 15% SDS-PAGE and visualized by Coomassie brilliant blue R-250 staining. The protein concentration was quantified with an Enhanced BCA protein assay kit (Beyotime, Shanghai, China).

### 4.6. Generation of Mouse Polyclonal Antibody Against rCsCD209

Polyclonal antibody against rCsCD209 was prepared as follows. rCsCD209 (50 μg) was emulsified with 50 μL complete Freund’s adjuvant (Sigma, St. Louis, MO, USA), and injected into mice of about 6 weeks old mice by multipoint subcutaneous injection. The second and third subcutaneous injection were performed on the 16th and 30th day with Freund’s incomplete adjuvant. The fourth inoculation was performed on 37th day without any adjuvant. Serum was collected from the blood samples at one week after the fourth subcutaneous injection. The serum antibody titer of the polyclonal antibody against CsCD209 was determined by ELISA as reported previously [46]. The serum was then buffered by PBS and loaded onto a 2-mL protein A column (GE Healthcare, Piscataway, NJ, USA). After PBS washing, immunoglobulin G (IgG) was eluted with 100 mM glycine-HCl (pH 2.8). The eluate was rapidly neutralized with 1 M Tris-HCl (pH 8.5), and dialyzed extensively against PBS (pH 7.2) overnight. The eluted IgG was concentrated through a 10 kDa cut-off filter (Millipore, Bedford, MA, USA) by centrifugation at 5000 rpm for 30 min, and the concentration of IgG was quantified to be 0.72 mg/mL. The binding specificity of the purified antibody towards CsCD209 was determined by Western blotting.

### 4.7. Western Blot Analysis

#### 4.7.1. Western Blot Analysis of CsCD209 in Head Kidney Leukocytes and Serum

The head kidney was manually homogenized in radioimmunoprecipitation assay (RIPA) buffer (Sigma, St. Louis, MO, USA) and lysed on ice for 20 min. After centrifugation at 4 °C at 12000 rpm for 10 min, the supernatant was collected, and the protein concentration was quantified with the Enhanced BCA protein assay kit (Beyotime, Shanghai, China). Blood was drawn from the caudal vein of tongue sole, and serum was prepared as described previously [43]. Head kidney leukocyte protein (50 μg) and serum protein (100 μg) were mixed with SDS-PAGE loading buffer, boiled at 100 °C for 10 min and then separated by SDS-PAGE. The protein bands were transferred from gel onto a polyvinylidene fluoride (PVDF) membrane, and the PVDF membrane was soaked in blocking buffer (5% BSA and 0.05% Tween 20 of PBS, pH 7.2) at room temperature for 1 h. The membrane was then incubated with antibody against rCsCD209 (1:1000 dilution) at room temperature for 1 h followed by extensive washing. The membrane was further incubated with horseradish peroxidase (HRP) conjugated goat anti-mouse IgG (Abcam, Cambridge, Cambridgeshire, UK, 1:5000 dilution) at room temperature for 1 h. After extensive washing, the immune-reactive protein bands were visualized by using an enhanced chemiluminescence kit (Pierce, Rockford, IL, USA).

#### 4.7.2. Western Blot Analysis of rCsCD209 Binding to Microbes

rCsCD209 or rTrx were incubated with microbes (1×10^9^ cells/mL) at a final concentration of 100 μg/mL at room temperature for 1 h with continuous rotation. The microbes were then washed three times with PBS and suspended in SDS-PAGE loading buffer. After boiling at 100 °C for 10 min, the samples were separated by SDS-PAGE. The proteins were transferred from gel onto a PVDF membrane, and the PVDF membrane was soaked in blocking buffer (5% BSA and 0.05% Tween 20 of PBS, pH 7.2) at room temperature for 1 h. The membrane was then incubated with monoclonal antibody against His tag (Abcam, Cambridge, Cambridgeshire, UK, 1:1000 dilution) at 4 °C overnight followed by extensive washing. The membrane was further incubated with HRP conjugated goat anti-mouse IgG (1:5000 dilution) at room temperature for 1 h. After extensive washing, the immuno-blotted protein bands were visualized by using an enhanced chemiluminescence kit.

### 4.8. Flow Cytometry

#### 4.8.1. Flow Cytometry Analysis of CsCD209 Expression

Tongue sole head kidney was surgically removed and passed through a nylon mesh with L15 medium (Gibco, Grand Island, NY, USA). The cell suspension was then placed on the top of a 61% Percoll (Pharmacia, Uppsala, Sweden) gradient and centrifuged at 4000 rpm for 10 min at 4 °C. Leukocytes were collected from the interphase, and the remaining red blood cells were removed as previously reported [47]. The cell viability of head kidney leukocytes was measured using the trypan blue (Sigma) exclusion assay, and the percentage of live cells was more than 95% of the total leukocytes. The cells (1 × 10^6^ leukocytes) were incubated with 5% bovine serum albumin (BSA) at 22 °C for 1 h followed by PBS washing for three times. Antibody against rCsCD209 (1:1000 dilution) was added to the cells, followed by incubation at 22 °C for 1 h. After washing three times with PBS, the cells were incubated with Alexa Fluor 488-labeled Goat anti-mouse IgG (Abcam, 1:1000 dilution) at 22 °C for 1 h. The cells were washed as above and determined for fluorescence intensity with a FACSAria II flow cytometer (BD Biosciences, Heidelberg, Germany). The experiment was performed in triplicate.

#### 4.8.2. Flow Cytometry Analysis of rCsCD209 Binding to Bacteria

rCsCD209 (10 μg/mL) was preincubated with or without mannose (200 mM) or mannan (100 μg/mL) at room temperature for 1 h with continuous rotation, and further mixed with *E. tarda* (1 × 10^8^ cells/mL) for 30 min with continuous rotation. The bacteria were then washed three times with PBS, and incubated with FITC-labeled anti-His antibody (Abcam, Cambridge, Cambridgeshire, UK, 1:500 dilution) for another 30 min. Flow cytometry was performed to determine the binding activity of rCsCD209 to bacteria after extensive washing.

#### 4.8.3. Flow Cytometry Analysis of Phagocytosis

*E. tarda* was washed by PBS (pH 7.2) and then incubated with 0.1 mg/mL FITC (Sigma, St. Louis, MO, USA) at room temperature with gentle stirring for 1 h. The FITC-labeled *E. tarda* was extensively washed with PBS and incubated with or without rCsCD209 (100 μg/mL) at room temperature for 1 h. After washing with PBS for three times, FITC-labeled *E. tarda* was added to head kidney leukocytes (1 × 10^6^ cells/mL) at a ratio of 10:1 and incubated for 1 h at room temperature. The cells were washed with PBS for three times, and trypan blue (1.2 mg/mL) was added to the cells to quench surface-bound FITC-labeled bacteria. The phagocytic percentage was analyzed by flow cytometry (BD Biosciences, Heidelberg, Germany). The phagocytic index was calculated as the percentage of phagocytic leukocytes multiplied by the mean intensity of that population [48]. The experiment was performed in triplicate.

### 4.9. Microscopic Analysis of the Subcellular Distribution of CsCD209 

Head kidney leukocytes prepared as above were plated on glass-bottomed culture dishes and incubated at 22 °C for 3 h before use. The cells were then incubated with 4% paraformaldehyde at room temperature for 15 min and further incubated with 0.1% Triton X-100 for 10 min. After washing with PBS, 3% BSA in PBS was added to block non-specific binding sites for 1 h. The cells were incubated with anti-rCsCD209 antibody (1: 1000 dilution) at room temperature for 1 h, and washed with PBS for three times. Alexa Fluor 594-labeled goat anti-mouse IgG (1:1000 dilution) was added to the cells and incubated at room temperature for 1 h. The cells were further incubated with DiO for 30 min and then with 4′-6-diamidino-2-phenylindole (DAPI) for 5 min. After extensive washing with PBS, the cells were monitored with a Carl Zeiss LSM 710 confocal microscope (Carl Zeiss, Jena, Germany).

### 4.10. Statistical Analysis

All experiments were repeated three times. The two-sample Student’s *t* test was used for the comparisons between groups. Statistical analysis was performed with GraphPad Prism 5 software (GraphPad Software, La Jolla, CA, USA). Results are shown as means ± SEM, and statistical significance was defined as *p* < 0.05.

## Supplementary Materials

Supplementary materials can be found at www.mdpi.com/1422-0067/18/9/1848/s1.
