# Supplementary material for: Tongue Sole CD209: A Pattern-Recognition Receptor that Binds a Broad Range of Microbes and Promotes Phagocytosis
Source: Int J Mol Sci. 2017 Sep 4;18(9):1848. doi: 10.3390/ijms18091848 (PMC5618497; doi:10.3390/ijms18091848)
Supplement: Supplementary file 1 [file ijms-18-01848-s001.pdf]

Supplementary Figures

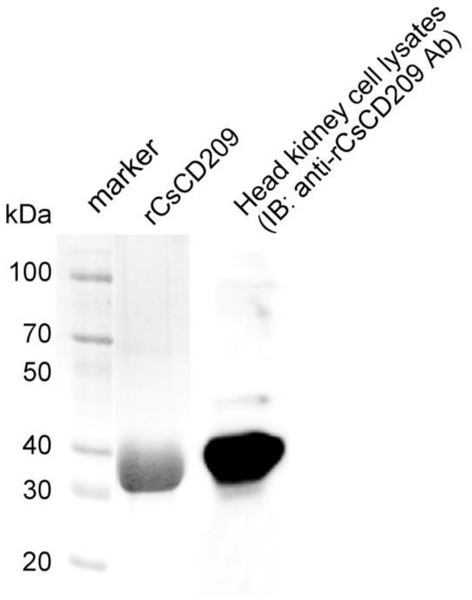

Figure S1. SDS-PAGE analysis of purified rCsCD209 and Western blot analysis of anti-rCsCD209 antibodies. rCsCD209 was expressed and purified by Ni-NTA chromatography, and further separated by SDS-PAGE. Western blot was performed to detect the CsCD209 in head kindney leukocytes by polyclonal antibodies against rCsCD209.

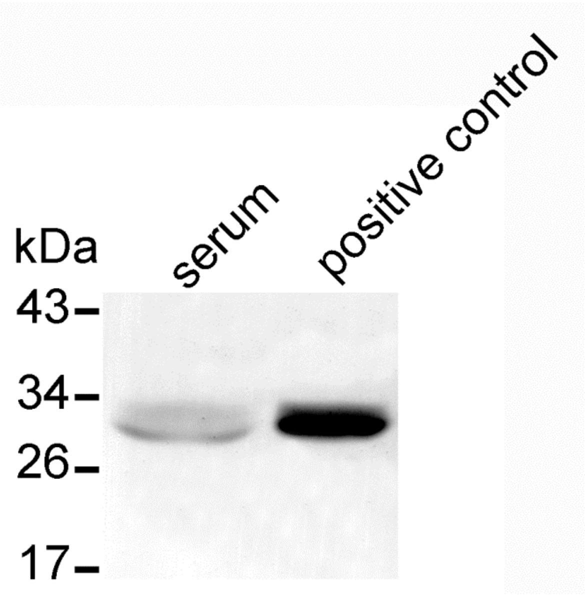

Figure S2. The soluble CsCD209 examined by Western blot. Serum protein was separated by SDS-PAGE, transferred to Polyvinylidene fluoride (PVDF) membrane and immunoblotted by anti-rCsCD209 antibody. Head kidney leukocyte proteins were used as a positive control.

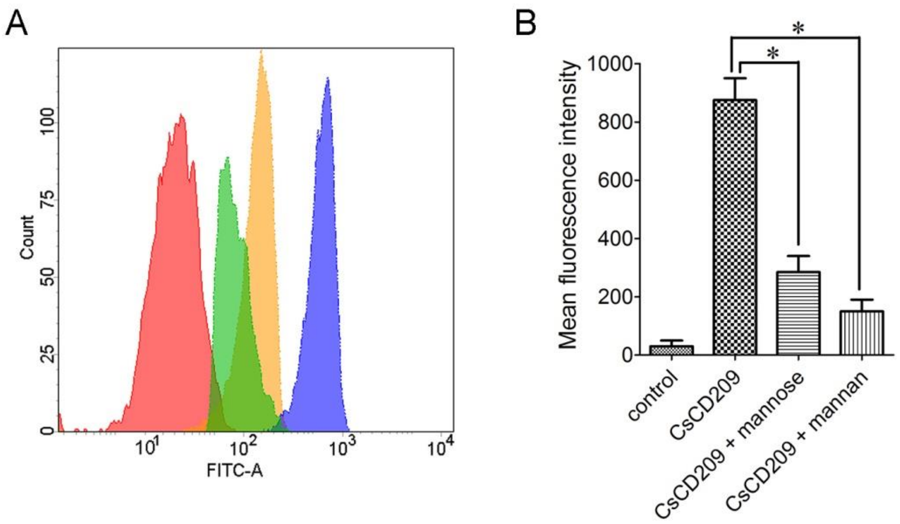

Figure S3. Effects of mannose and mannan on rCsCD209 binding to bacteria. (**A**) rCsCD209 was preincubated with or without mannose or mannan before incubating with *Edwardsiella tarda*. The cells were treated with FITC-labeled anti-His antibody, and rCsCD209-bacteria binding was determined by flow cytometry. Red histogram: control; blue histogram: rCsCD209; yellow histogram: $rCsCD209^{+}$ mannose; green histogram: $rCsCD209^{+}$ mannan. (**B**) The mean fluoresence intensity in (**A**) was statistically calculated. Results are means ± SEM (n = 3), * $p < 0.05$.
